# Novel kinetoplastid-specific cAMP binding proteins identified by RNAi screening for cAMP resistance in *Trypanosoma brucei*

**DOI:** 10.3389/fcimb.2023.1204707

**Published:** 2023-07-05

**Authors:** Sabine Bachmaier, Matthew K. Gould, Eleni Polatoglou, Radoslaw Omelianczyk, Ana E. Brennand, Maha A. Aloraini, Jane C. Munday, David Horn, Michael Boshart, Harry P. de Koning

**Affiliations:** ^1^ Faculty of Biology, Genetics, Ludwig-Maximillians University Munich (LMU), Martinsried, Germany; ^2^ Institute of Infection, Immunity and Inflammation, College of Medical, Veterinary and Life Sciences, University of Glasgow, Glasgow, United Kingdom; ^3^ The Wellcome Centre for Anti-Infectives Research, School of Life Sciences, University of Dundee, Dundee, United Kingdom

**Keywords:** *Trypanosoma brucei*, cAMP, CARP, flagellum, adenylate cyclase, RNAi screening, NPD-001

## Abstract

Cyclic AMP signalling in trypanosomes differs from most eukaryotes due to absence of known cAMP effectors and cAMP independence of PKA. We have previously identified four genes from a genome-wide RNAi screen for resistance to the cAMP phosphodiesterase (PDE) inhibitor NPD-001. The genes were named cAMP Response Protein (CARP) 1 through 4. Here, we report an additional six CARP candidate genes from the original sample, after deep sequencing of the RNA interference target pool retrieved after NPD-001 selection (RIT-seq). The resistance phenotypes were confirmed by individual RNAi knockdown. Highest level of resistance to NPD-001, approximately 17-fold, was seen for knockdown of CARP7 (Tb927.7.4510). CARP1 and CARP11 contain predicted cyclic AMP binding domains and bind cAMP as evidenced by capture and competition on immobilised cAMP. CARP orthologues are strongly enriched in kinetoplastid species, and CARP3 and CARP11 are unique to *Trypanosoma*. Localization data and/or domain architecture of all CARPs predict association with the *T. brucei* flagellum. This suggests a crucial role of cAMP in flagellar function, in line with the cell division phenotype caused by high cAMP and the known role of the flagellum for cytokinesis. The CARP collection is a resource for discovery of unusual cAMP pathways and flagellar biology.

## 1 Introduction

Conservation of signalling pathways and proteins among different phyla of eukaryotes is very limited, particularly in protozoa. Cyclic nucleotides are present as second messengers in almost all organisms, and in protozoa they can regulate growth, development and metabolic adaptation. In trypanosomes an important role for cAMP is suggested by an estimated 80 adenylate cyclase encoding genes (Salmon et al., 2012b), many of which are expressed throughout the life cycle (Alexandre et al., 1996); multiple are present in the plasma membrane (Bridges et al., 2008) or mainly localised to the flagellar surface (Paindavoine et al., 1992; Saada et al., 2014). These cyclases consist of a conserved catalytic domain, a single trans-membrane domain and a variable extracellular domain (Salmon, 2018). It is thus possible that various extracellular ligands control their activity (Paindavoine et al., 1992), but none have been identified and activation by dimerization has been observed under acidic, hypotonic or other stress conditions (Nolan et al., 2000; Gould and de Koning, 2011; Salmon, 2018). In recent years, the adenylate cyclases have been implicated in cytokinesis (Salmon et al., 2012a), immune evasion in the mammalian host (Salmon et al., 2012b), and social motility in the procyclic insect stage of the parasite (Lopez et al., 2015; Oberholzer et al., 2015; Saada et al., 2015). Control of cAMP homeostasis is crucial as knockdown of the locus containing the cAMP phosphodiesterases TbrPDEB1 and TbrPDEB2 is lethal (Oberholzer et al., 2007) and inhibitors of these enzymes have potent antitrypanosomal activity (de Koning et al., 2012; de Heuvel et al., 2019). However, the effectors and cascades activated or inhibited by cAMP remain largely unknown in trypanosomes, given the absence of genes encoding the known cAMP effectors in mammalian cells. Most importantly, the homologue of Protein Kinase A (PKA) is not activated by, and does not bind, cyclic nucleotides in *T. brucei* (Bachmaier and Boshart, 2014; Bubis et al., 2018; Bachmaier et al., 2019). This has stimulated a screen aiming at identification of novel cAMP effectors or target proteins. We previously reported a set of four *T. b. brucei* genes whose RNAi repression protected against the high intracellular cAMP concentration resulting from the inhibition of TbrPDEB1/2; these genes were termed cAMP Response Protein (CARP) 1 – 4 (Gould et al., 2013). Out of these, only CARP3, a gene unique to trypanosomes, has been investigated and it was shown to be essential for social motility of procyclic trypanosomes through direct interaction with and regulation of adenylate cyclases (Bachmaier et al., 2022). CARP3 is therefore an upstream regulator of cAMP signalling and the effectors and cAMP-binding protein(s) remain to be identified.

The *Trypanosoma brucei* subspecies *T. b. gambiense* and *T. b. rhodesiense* are responsible for the disease known as Human African Trypanosomiasis (HAT), or sleeping sickness (Büscher et al., 2017), whereas *T. b. brucei* and the related trypanosomes *T. congolense*, *T. vivax*, *T. evansi* and *T. equiperdum* all contribute to the various manifestations of Animal African Trypanosomiasis (AAT), known under names such as nagana, surra and dourine (Giordani et al., 2016). Sleeping sickness has long been among the most neglected diseases, being transmitted by the tsetse fly, which makes it a problem of rural Africa, but more recently progress has been made and case numbers have dropped with more active control measures (Barrett, 2018; Franco et al., 2020) and the introduction of the first oral drug, fexinidazole, against the infection (Lindner et al., 2020). In contrast, little progress has been made in reducing the impact of AAT, which continues to have devastating effects on livestock and, consequently, on rural economies and food security in Africa and beyond, in part because *T. evansi*, *T. vivax* and *T. equiperdum* are not dependent on tsetse fly transmission (Jones and Dávila, 2001; Desquesnes, 2004; Desquesnes et al., 2013; Aregawi et al., 2019). No new animal trypanosomiasis drugs have been introduced for decades and the treatment options are both limited and threatened by resistance (Delespaux and de Koning, 2007; Melaku and Birasa, 2013; Giordani et al., 2016; Kasozi et al., 2022). Among the issues that have held back drug development against trypanosomiasis is the paucity of well-validated drug targets – a result of the many gaps in our knowledge of their unique biochemistry and cell biology. The limited understanding of mostly non-conserved signalling mechanisms and proteins is one reason why these pathways, preferred targets in mammalian drug development, are less exploited for the parasites.

Here we revisit the screen that identified CARP1 – 4 by deep sequencing of the RNAi library grown out after challenge with PDE inhibitor NPD-001 (Gould et al., 2013). We identify six additional genes that confer resistance to perturbation of intracellular cAMP, CARP6 – CARP11, and validate their phenotype by targeted RNAi for each individual gene. Most of the CARPs are unique to the kinetoplastidae. CARP1 and CARP11 contain potential cAMP-binding domains and cAMP binding to these proteins was confirmed by capture on cAMP-linked agarose beads. The identification of novel and trypanosomatid-specific cAMP effector candidates will elucidate the evolution of cAMP signalling and eventually provide attractive drug target candidates.

## 2 Methods

### 2.1 Trypanosome culture conditions

Bloodstream forms of the monomorphic *Trypanosoma brucei brucei* strain Lister 427 MiTat 1.2 were cultivated at 37°C and 5% CO_2_ in modified HMI-9 medium (Vassella et al., 1997) supplemented with 10% (v/v) heat-inactivated foetal bovine serum (FBS). For maintenance of tetracycline repressor Tet^R^ and T7 polymerase in the trypanosome cell lines MiTat 1.2 13-90 or MiTat 1.2 single marker (Wirtz et al., 1999), 2.5 µg/mL G418 and 5 µg/mL hygromycin B or only 2.5 µg/mL G418, respectively, were added to the culture medium. Cell density was monitored using a haemocytometer and was kept below 1×10^6^ cells/mL for continuous growth of replicative long slender bloodstream forms.

### 2.2 RNAi target sequencing

Sequencing of the libraries was performed on a Thermo Scientific Ion Proton at the Glasgow Polyomics facility using the 200 base pair sequencing kit. Sequences containing a terminal RNAi-vector junction sequence (GCCTCGCGA) were mapped to the *T. brucei* 927 reference genome (Alsford et al., 2012), the selection allowed for a single aberration, either a base change or a single base insertion or deletion. The filtered reads were mapped to the reference genome with Bowtie2 using the local mode alignment. The aligned reads were then assigned to a region of interest, reads that either were fully contained within the region or only partially overlap the region of interest. The percentage of mapped reads for any gene is thus the number of reads aligned to that gene, divided by the total of all reads × 100. The counts for each replicate were also expressed as normalised mapped reads by dividing the total assigned to any gene by the CDS length × 100.

### 2.3 Cloning and generation of transgenic trypanosomes

#### 2.3.1 In situ tagging of CARP6 and CARP10

For *in situ* tagging of CARP6 and CARP10, the long primer PCR tagging strategy was used on pPOTv2 as a template (Dean et al., 2017) using primers 2CTb427.10.12390F (CGGCATTTGAGGAGATTGAGAGACGACGGCAGCAGGAAGCTGCAGCAAAGGCCGCTGCGGACGATGCTATGCCGTTAGTAactagtgtgagcaagg) and 2CTb427.10.12390R (AAAAAAAAAAGTTAGGGGACCGCGAAGGAAAAAAAGGAGTAAAGAACCAGGTCACTCCTGAATATGTACATGGTAGAGATccaatttgagagacctgtgc) for C-terminal YFP-TY (Yellow Fluorescent Protein) tagging of CARP6 and primers 2NTb427.02.5030F (TGGTGTTTTGAGTGCAATGTTTTTTTCACTTCTTTTTCTTTTCTTTACCACTTTAGCCGGAAGTTGTTGGCGGTGTCGCGgtataatgcagacctgctgc) and 2NTb427.02.5030R (ACCGTGTTACTCGACACATGATCGGTACACACAGCCTTCAAATATCTGTAAATCTGCACCACTGCTTCCTTCGTTGTCATcttgtacagctcgtccatgc) for N-terminal TY-YFP tagging of CARP10. PCR products were purified by phenol-chloroform extraction prior to transfection and transfected cells were grown in the presence of 2 µg/mL blasticidin or hygromycin, respectively.

#### 2.3.2 Generation of inducible CARP knock down cell lines

For tetracycline-inducible knock down of putative CARPs, the following fragments were amplified from *T. brucei* Lister 427 MiTat 1.2 single marker genomic DNA and cloned into p2T7-177-BLE (Wickstead et al., 2002) *via* BamHI and XhoI restriction sites: *CARP5* (Tb927.10.1740): RNAi targeting fragment: ORF nt 365-776, amplified with primers Tb427.10.1740_RNAi_Frag-F and Tb427.10.1740_RNAi_Frag-R; *CARP6* (Tb927.10.12390): RNAi targeting fragment: ORF nt 256-682, amplified with primers Tb427.10.12390_RNAi_Frag-F and Tb427.10.12390_RNAi_Frag-R; *CARP7* (Tb927.7.4510): RNAi targeting fragment: ORF nt 625-1177, amplified with primers Tb427.07.4510_RNAi_Frag-F and Tb427.07.4510_RNAi_Frag-R; *CARP8* (Tb927.11.3910): RNAi targeting fragment: ORF nt 2076-2624, amplified with primers Tb427tmp.02.1410_RNAi_Frag-F and Tb427tmp.02.1410_RNAi_Frag-R; *CARP9* (Tb927.8.4640, alternative name: CMF19, component of motile flagella 19): RNAi targeting fragment: ORF nt 601-1092, amplified with primers Tb427.08.4640_RNAi_Frag-F and Tb427.08.4640_RNAi_Frag-R; *CARP10* (Tb927.11.7180): RNAi targeting fragment: ORF nt 2-439, amplified with primers Tb427tmp.02.5030_RNAi_Frag-F and Tb427tmp.02.5030_RNAi_Frag-R; *CARP11* (Tb927.7.2320): RNAi targeting fragment: ORF nt 524-990, amplified with primers Tb427.07.2320_RNAi_Frag-F and Tb427.07.2320_RNAi_Frag-R. The RNAi plasmids were linearized with NotI for transfection and cells were selected by addition of 2.5 µg/mL phleomycin to the culture medium.

#### 2.3.3 Inducible overexpression of CARP1

The CARP1 ORF was amplified by PCR from *T. brucei* MiTat 1.2 genomic DNA using primers Tb11.01.7890 SfiI up and Tb11.01.7890 BamHI low and cloned into plew82 (Wirtz et al., 1999) *via* SfiI and BamHI. Point mutations in the predicted CARP1 cyclic nucleotide binding domains were generated by site-directed mutagenesis using primers Tb11.01.7890 CNBDmut1 up and Tb11.01.7890 CNBDmut1 low (R320L, AGA to TTA), Tb11.01.7890 CNBDmut2 up and Tb11.01.7890 CNBDmut2 low (R458L, CGA to CTA), or Tb11.01.7890 CNBDmut3_R605L up and Tb11.01.7890 CNBDmut3_R605L low (R605L, AGG to CTG), respectively. The plasmids were linearized with NotI for transfection and cells were grown in presence of 2 µg/mL blasticidin.

### 2.4 Recombinant protein expression in *Escherichia coli*

The CARP11 ORF (Tb927.7.2320) was amplified from *T. brucei* MiTat 1.2 genomic DNA with primers Tb927.7.2320_SUMO_BamHI_fw (GCACTAGGATCCATGAGCATCGTCAATCAG) and Tb927.7.2320_SUMO_HindIII_rev (CCACCAAGCTTTCACTTGACACGACTGCA) and cloned into pETM11_SUMO3 *via* BamHI and HindIII. The protein was expressed in *E. coli* Rosetta (grown in TB with 1 M sorbitol, overnight induction of protein expression with 200 µM IPTG at 16°C) and the soluble fraction was used for pull-down with cAMP agarose.

### 2.5 Generation of polyclonal antibodies

The *CARP1* ORF was amplified using primers Tb427tmp.01.7890.fl.f.10His (ataccatgggccaccaccaccaccaccaccaccaccaccacggcgcgggcGGTAGTTATGAATACCCAGACTAC) and Tb427tmp.01.7890.fl.b (aattcggatcctggctTTACCTTTTCGCCATGAACTCTTT) introducing an N-terminal His_10_ tag and cloned into pETDuet-1 *via* NcoI and BamHI restriction sites. The protein was expressed in *E. coli* Rosetta and purified under denaturing conditions using Ni-NTA columns. After separation of the concentrated protein fractions on a 10% SDS gel, rabbits were immunized with Coomassie-stained CARP1-containing gel slices according to a standard immunization protocol (Pineda, Berlin). The CARP1 antiserum was affinity-purified using His_10_-CARP1 according to the method of Olmsted. (Olmsted, 1981).

### 2.6 Pull-down with cAMP agarose

1×10^8^ *T. brucei* cells overexpressing CARP1 were washed twice with PBS and lysed in 300 µL lysis buffer (50 mM Tris-HCl pH 7.5, 150 mM NaCl, 2 mM EGTA, 0.2% NP-40, Roche Complete protease inhibitor) for 20 min at 4°C. For *E. coli* lysates, 12.5 mL of a logarithmically growing culture expressing His_6_-SUMO-CARP11 was lysed in 500 µL lysis buffer (50 mM Tris-HCl pH 7.5, 150 mM NaCl, 1% Triton X-100, 570 µM PMSF) for 10 min at 4°C with sonication (Bioruptor, Diagenode, 10 min on ice, 30 s on/off, high power). Soluble lysates were incubated with plain agarose beads (Biolog Bremen) for 30-60 min at 4°C to remove proteins binding non-specifically to the bead matrix. Pull-downs were performed by incubation of the pre-cleared lysates (= input fractions) with 30 µL (CARP1) or 40 µL (CARP11) 2-AHA- or 8-AHA-agarose (Biolog Bremen Cat. No. A054, A028) beads slurry for 30 min – 2 h at 4°C, followed by four washes with lysis buffer. Bound proteins were eluted by boiling (5 min, 95°C) with 20 µL (CARP1) or 40 µL (CARP11) 2× Laemmli sample buffer. Equal volumes of input and bound fractions were analysed by Western blot, giving an enrichment factor in the bound fraction versus the input of 15× for CARP1 (300 µL/20 µL) and 12.5× for CARP11 (500 µL/40 µL).

### 2.7 Dose-response cell viability assay

NPD-001 sensitivity of the inducible CARP knock down cell lines was assessed using the Alamar blue (resazurin) cell viability assay exactly as described previously (Gould et al., 2013).

### 2.8 Western blot

Western blot analysis was performed as previously described (Salmon et al., 2012a) with modifications for detection of CARP1. Briefly, lysates of 3-5×10^6^ trypanosomes were separated on 10% polyacrylamide gels, transferred to a PVDF membrane *via* semi-dry blotting and blocked with Kem-En-Tec synthetic blocking buffer for 1 h at room temperature. The blots were incubated with primary antibodies (rabbit anti-CARP1, 1:1000; mouse anti-PFR-A/C [(Kohl et al., 1999), 1:2000; PFR, paraflagellar rod protein)] overnight at 4°C, followed by secondary antibody (IRDye680LT anti-rabbit and IRDye800CW anti-mouse, LI-COR, both 1:5000) detection for 1.5 h at room temperature. For detection of His-tagged proteins expressed in *E. coli*, proteins were blotted onto PVDF membranes and blocked with 5% milk for 1 h at room temperature, followed by incubation with mouse anti-His (1:1000, BioRad MCA1396GA) overnight at 4°C and secondary antibody detection with IRDye800CW anti-mouse (1:5000, LI-COR).

### 2.9 Bioinformatics

CARP sequences were retrieved from TriTrypDB release 58 (https://tritrypdb.org/tritrypdb/app), information on orthology was obtained from orthoMCL release 6.11 (https://orthomcl.org/orthomcl/app), and domain predictions were done with Superfamily 2 (https://supfam.mrc-lmb.cam.ac.uk/SUPERFAMILY/), Pfam version 35.0 (http://pfam.xfam.org/), and/or SMART (http://smart.embl-heidelberg.de/). AlphaFold2 structure predictions for CARP1 and CARP11 were retrieved from http://wheelerlab.net/alphafold/ and cyclic nucleotide binding domains predicted by Superfamily 2 (https://supfam.mrc-lmb.cam.ac.uk/SUPERFAMILY/) were superimposed with crystal structures of the known cAMP binding proteins *E. coli* CRP (PDB 4N9H) and *Bos taurus* PKARIa (PDB 1RGS) using Pymol (*The PyMOL Molecular Graphics System, version 2.5* Schrödinger, LLC). Predicted CNBD phosphate binding cassette sequences of CARP1 and CARP11 were aligned with those of *E. coli* CRP, *Mus musculus* Epac4 and *Bos taurus* PKARIa using CLC Main Workbench version 21.0.5 (Qiagen). For structure similarity searches, the AlphaFold ID was obtained from TriTrypDB release 63 or directly from wheelerlab.net and the structure downloaded as a PDB file from https://alphafold.ebi.ac.uk/, which was then used to run a structure search on the Dali server (http://ekhidna2.biocenter.helsinki.fi/dali/). The top hit of the resulting output was then viewed in the RSCB protein database (PDB; https://www.rcsb.org/).

## 3 Results

### 3.1 RNA-interference target sequencing after selection for resistance to NPD-001

As described by Gould et al. (2013), a genome-wide RNAi library screen was carried out with bloodstream *T. b. brucei* under selection pressure with the phosphodiesterase inhibitor NPD-001, previously known as CpdA (de Koning et al., 2012), following established protocols (Alsford et al., 2011; Alsford et al., 2012). In the initial report, we identified four genes by sequencing PCR-fragments from amplified prominent bands on an agarose gel and designated them CARP1 – 4. The sample from this selection has now been subjected to RIT-seq deep sequencing, identifying the RNAi sequences and mapping them to the genome. This yielded a far more diverse and quantitative data set of genes that, upon RNAi knockdown, protect to any degree against the strongly elevated levels of intracellular cAMP resulting from the treatment with NPD-001. A total of 1183 genes was included in this dataset, with raw total counts between 74,563,689 and 1, out of a total of 105,662,532 bases mapped to CDS. Among the most frequent hits were CARP1 – CARP4 and an additional 7 genes were selected from the genes with highest counts, and designated CARP5 - CARP11 (**Table 1**). In addition, four adenylate cyclase-encoding genes were identified with high abundance, consistent with cyclase knockdown causing a reduction of cAMP levels (Salmon et al., 2012a).

**Table 1:** Summary of cAMP response genes (CARPs) conferring cAMP sensitivity.

Name	Tb427 accession^1^	Tb927 accession^1^	Tb927 ORF length (a.a.)	% of mapped RIT-seq bases	Normalised mapped reads	RNAi targeting region (nt)
CARP1	Tb427_110181700	Tb927.11.16210	705	70.6	35188	Gould et al., 2013
CARP2	Tb427_110145400	Tb927.11.12860	302	0.42	492	Gould et al., 2013
CARP3	Tb427_070059100	Tb927.7.5340	498	2.4	1704	Gould et al., 2013
CARP4	Tb427_030010200	Tb927.3.1060	779	13.9	18882	Gould et al., 2013
CARP5	Tb427_100020600	Tb927.10.1740	382	3.38	3108	365-776
CARP6	Tb427_100131300	Tb927.10.12390	258	4.7	6374	256-682
CARP7	Tb427_070050500	Tb927.7.4510	407	0.092	79	625-1177
CARP8	Tb427_110044600	Tb927.11.3910	1108	1.1	339	2076-2624
CARP9	Tb427_080051400	Tb927.8.4640	385	0.25	228	601-1092
CARP10	Tb427_110076700	Tb927.11.7180	653	0.34	180	2-439
CARP11	Tb427_070027800	Tb927.7.2320	605	0.046	27	524-990

^1^Gene IDs are from TriTrypDB.org.

**Figure 1:**
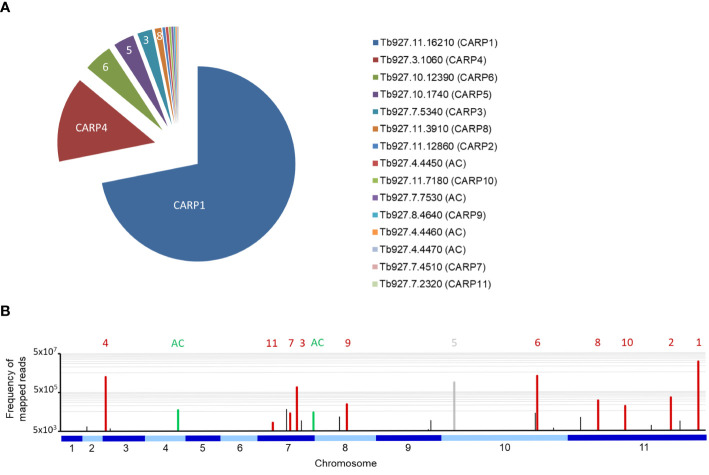
**(A)** Pie chart of the percentage of all reads mapped to each of the eleven CARP genes and to the four GRESAG4 adenylate cyclases (AC). The gene identifiers from TriTrypDB and the CARP numbering are indicated. **(B)** Frequency of mapped RIT-seq reads indicated on a genome map of *T. brucei* 927. Numbered CARPs are indicated by red bars and the corresponding numbers above them; only CARP5 is indicated in grey to indicate this was a false positive hit. Adenylate cyclases (AC) are similarly indicated in green; The green bar on chromosome 4 is an amalgamation of three bars, for genes Tb927.7.4450, Tb927.7.4460 and Tb927.7.4470. Black bars represent genes that were not followed up for validation by targeted RNAi for this study.

**Figure 1** shows a pie-chart representation of the % of total mapped reads for each of the CARPs and adenylate cyclases that together account for 98.2% of all mapped reads in the RIT-seq analysis. Clearly, the most frequent gene targeted was CARP1, with 70.6% of all mapped reads, followed by CARP4 with 13.9%.

Of the newly identified CARPs, CARP6 was the most-targeted gene and CARP11 the least targeted, accounting for 4.69% and 0.046% of mapped reads, respectively. The four listed adenylate cyclases were identified between 0.42% and 0.14% of mapped reads. A genome map of RIT-seq hits is depicted in **Figure 1**, which shows the designated CARPs, as well as the positions of the adenylate cyclases and other ‘hits’ that were not taken forward for verification by targeted RNAi. A listing of all 44 genes with normalised RIT-seq mapping ≥ 0.05% of mapped reads is given as **Supplemental Table S2**.

### 3.2 Phenotype confirmation of the new CARPs by RNAi of the individual genes

Previously, we confirmed that targeted RNAi knockdown of *CARP1 – CARP4* did confer protection to treatment with NPD-001, i.e. a shift to a significantly higher EC_50_ in our standardised resazurin-based drug sensitivity test. This induced resistance was specific, as it was not afforded to treatment with control drugs pentamidine, suramin and difluoromethylornithine (Gould et al., 2013).

RNAi target sequences were chosen for each of the genes *CARP5 – CARP11*, in order to obtain true independent confirmation. The RNAi target fragments (**Table 1**) were cloned into the expression vector p2T7-177-BLE (Wickstead et al., 2002) and transfected into the *T. b. brucei* cell line Lister 427 MiTat 1.2 13-90 (Wirtz et al., 1999) for tetracycline (TET)-inducible expression. As knockdown measured by mRNA levels does not necessarily correlate with protein levels in *T. brucei*, where the regulation is mostly at the level of translation rather than transcription (Clayton, 2016), protein levels for several of the CARPs were confirmed by Western blot using CARP1-specific antibodies (**Supplementary Figure S2A**) or antibodies against the TY tag *in situ* added to CARP6 and CARP10 (**Supplementary Figures S2B, C**). All three CARP proteins were strongly diminished after 24 h of TET induction. The knockdown of epitope-tagged CARP1–4 was also confirmed by Western blot in our previous paper (Gould et al., 2013).

**Figure 2:**
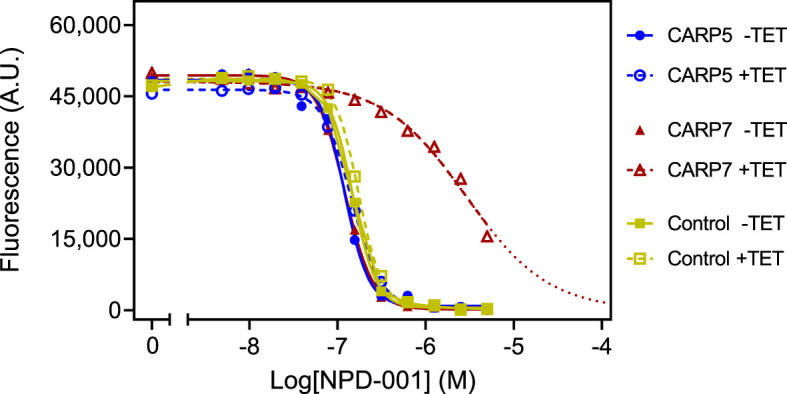
Representative graphs of the Alamar blue-based assay used to determine EC_50_ values for NPD-001. Dilution range for NPD-001 started at 5 μM and consisted of 11 doubling dilutions to 4.88 nM, and a no-drug control. The results shown are from a single experiment, representative of three independent replicates, performed with one of three clonal lines for each RNAi line (CARP5 clone 6 and CARP7 clone 4). Fluorescence read-outs in arbitrary units (A.U.), with background fluorescence subtracted, were plotted to a sigmoid curve with variable slope in Prism 9 to determine the EC_50_ values. Closed symbols and solid lines represent the control without tetracycline (TET), open symbols and dashed lines, with induction by TET. For the TET-induced CARP7 values the dotted line indicates extrapolation. Control cells were the parental Lister 427 ‘single marker’ strain derived from s427 by introducing the tetracycline repressor Tet^R^ and T7 polymerase (Wirtz et al., 1999).

EC_50_ values were obtained in parallel with and without TET induction, allowing statistical comparisons. Further, the NPD-001 sensitivity tests were performed with four biological replicates (independent transfectants) and each EC_50_ was obtained on at least three separate occasions. **Table 2** shows a summary of the CARP5 – CARP11 results, comparing the EC_50_ values obtained with and without TET induction and using the untransfected parental line as a further control. The RNAi knockdown resulted in significantly higher NPD-001 EC_50_ values for *CARP6 – CARP11*, but not for *CARP5*, which may therefore be classified as a false positive from the library screen. Indeed, further analysis showed this gene to be a frequent false hit in RIT-seq owing to the RNAi vector junction sequence GCCTCGCGA. Mis-priming from sequence GGGCCAGT within the Tb927.10.1740 (*CARP5*) CDS produced a 1245-bp product. For the confirmed CARPs 6–11, the level of protection against elevated cAMP by targeted RNAi was by far highest for *CARP7* (17-fold), whereas the targeted knockdown of the other genes typically resulted in < 4-fold resistance gain (**Table 2**). The difference in response to knockdown for CARP5 and the parental control versus CARP7 is illustrated in **Figure 2** with representative curves from the resazurin-based assays.

**Table 2:** Sensitivity to phosphodiesterase inhibitor NPD-001 upon inducible RNAi.

RNAi target	EC_50_ NPD-001 (Mean ± SEM), μM		Resistance factor	*P*-value
	- Tet	+ Tet		
parental control (n = 6)	0.14 ± 0.01	0.17 ± 0.01	1.18	0.0369
CARP5 (n = 9)	0.14 ± 0.01	0.15 ± 0.01	1.09	0.0988
CARP6 (n = 12)	0.25 ± 0.04	0.61 ± 0.02	2.41	<0.0001
CARP7 (n = 12)	0.22 ± 0.03	3.70 ± 0.27	16.91	<0.0001
CARP8 (n = 12)	0.21 ± 0.01	0.41 ± 0.02	1.98	<0.0001
CARP9 (n = 12)	0.26 ± 0.03	0.44 ± 0.08	1.72	0.0238
CARP10 (n = 9)	0.17 ± 0.01	0.30 ± 0.01	1.75	<0.0001
CARP11 (n = 12)	0.18 ± 0.01	0.30 ± 0.01	1.69	<0.0001

n = total number of replicates; P-value was calculated by unpaired, one-sided t-test.

### 3.3 Phylogeny and species distribution of CARP genes

**Figure 3:**
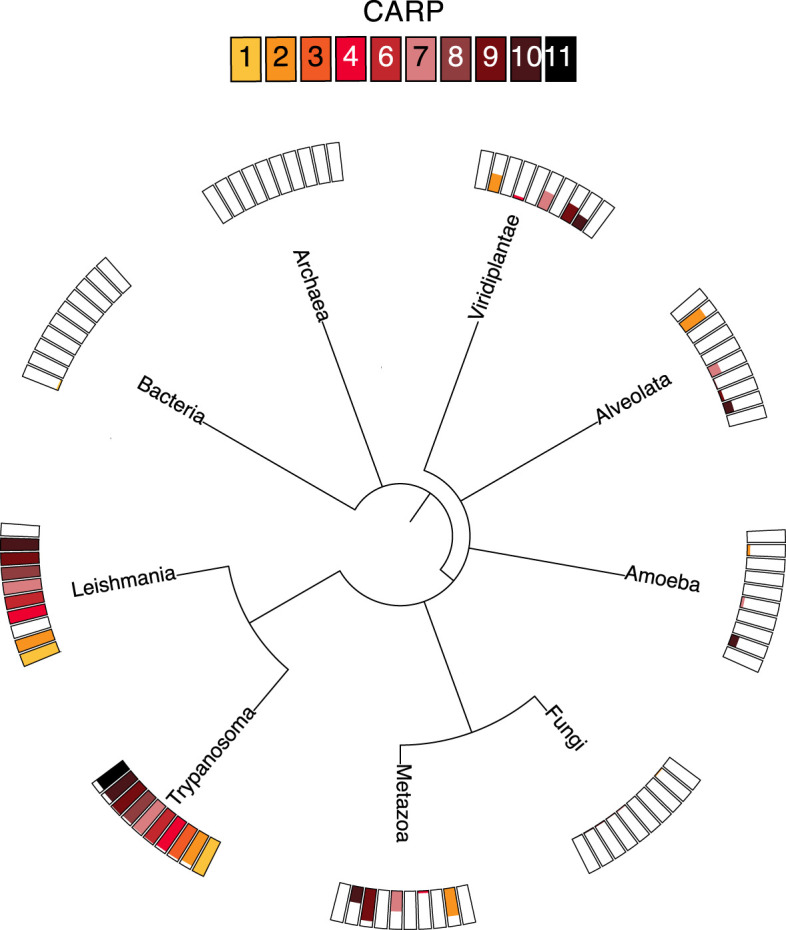
Distribution of ortholog groups for CARPs based on by OrthoMCL release 6.11 (https://orthomcl.org/orthomcl/app). Ortholog group IDs for CARPs were retrieved from TritrypDB release 58 (https://TritrypDB.org) and the tree was generated with phyloT version 2022.3 (https://phylot.biobyte.de/). Each CARP is shown by a differently coloured box and the size of the coloured bar represents the percentage of occurrence in the respective group of species.

CARP phylogeny was investigated by individual BLAST searches (blast.ncbi.nlm.nih.gov/Blast.cgi) as well as orthologue analysis based on the orthoMCL database (orthomcl.org) (**Figure 3**). Despite some small differences between the results of these two methods, all CARPs appear to be present in both African and American *Trypanosoma* species (*T. brucei*, *T. congolense*, *T. vivax*, *T. cruzi*) and most are also found in *Leishmania* genomes, except CARP3 and CARP11. CARP3 is also absent from the genomes of the insect gut parasites *Crithidia fasciculata* and *Angomonas deanei*, as well as the free-living trypanosomatid *Bodo saltans*. CARP11 is found in *A. deanei* and *B. saltans* but no orthologue could be identified in *C. fasciculata*.

Most CARPs are scarcely found outside the trypanosomatids, although there are some exceptions. CARP2 and CARP7 are found in some species of Viridiplantae, including the green algae *Chlamydomonas*, in some Alveolata, specifically the Apicomplexa, and in some metazoa including Mammalia; CARP9 is also present in *Chlamydomonas* and is quite widespread in metazoa, but is only found in very few Alveolata. Conversely, CARP4 is present in a few species of Viridiplantae and Metazoa but not in other non-trypanosomatids. Only CARP10 had a few orthologues in Amoebozoa and Alveolata and some in Viridiplantae and Metazoa. None of the CARPs had detectable orthologues in prokaryotes, fungi or in *Arabidopsis*. Overall, most CARPs are (almost) exclusive to trypanosomatids, with CARP3 limited to the genus *Trypanosoma*.

### 3.4 Analysis of domains architecture, structural similarity and subcellular localization of the CARP proteins

**Figure 4:**
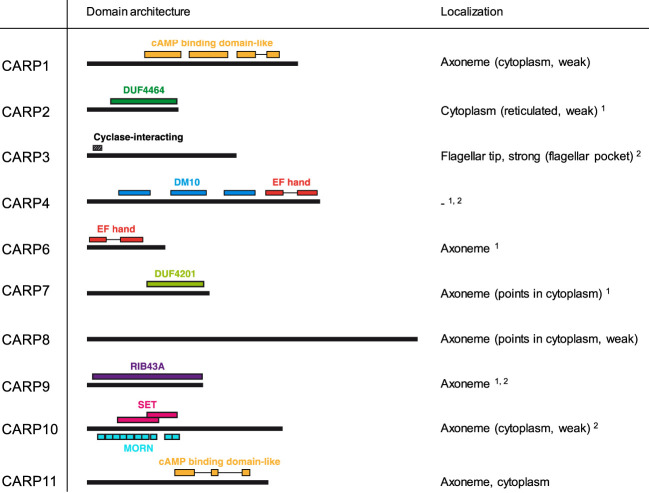
Domain predictions for CARPs by the domain profile databases Superfamily 2.0 (https://supfam.org/), Pfam version 35.0 (http://pfam.xfam.org/), and/or SMART (http://smart.embl-heidelberg.de/) are shown as schematic representation. CARP proteins are shown as black bars (to scale) and predicted domains are displayed by coloured boxes with IDs as indicated in the figure. For CARP3, the region required for flagellar tip localization and cyclase interaction (designated ‘Cyclase-interacting’) according to Bachmaier et al. (2022) is indicated. Subcellular localization of CARP proteins is given exactly as annotated in the TrypTag.org database (Dean et al., 2017). Additional evidence for axonemal^1^ or flagellar^2^ localization is taken from Broadhead et al. (2006) and Subota et al. (2014), respectively. DUF, domain of unknown function; MORN, Membrane Occupation and Recognition Nexus.

The domain structure and subcellular localization of the 10 confirmed CARPs (i.e., excluding CARP5) is summarised in **Figure 4**. Domains could be identified in profile database searches for all except CARP8, although some of these are Domains of Unknown Function (DUF), specifically DUF4464 as the main conserved domain in CARP2 and DUF4201 as the only domain detected in CARP7. Modelled structures were obtained from AlphaFold and used for structural similarity searches in the RCSB Protein Data Base (PDB); the top returns for each validated CARP are listed in **Supplemental Table S1**.

Strikingly, the TrypTag genome-wide tagging project (Dean et al., 2017; Billington et al., 2023) revealed an axonemal localization for 8/9 CARPs successfully tagged in procyclic *T. b. brucei*, which closely aligned with Gene Ontology (GO) assignments in TriTrypDB (**Supplemental Table S2**). The exception was CARP2, for which no GO assignments were available, and which showed a cytoplasmic distribution as N-terminal mNeonGreen fusion protein in TrypTag. However, CARP2 and CARPs 3–7, 9 and 10 are all present in at least one flagellar proteome of procyclic trypanosomes (Broadhead et al., 2006; Subota et al., 2014). Also, many CARP2 orthologues from other organisms are annotated as cilia- and flagella-associated proteins, and a bioinformatic study by Baron et al. (2007), identified CARP2 as a conserved component of motile cilia and flagella, supporting a flagellar localization. Structural similarity searches using AlphaFold and the Dali server highlighted as top hit a *Legionella pneumophilia* protein, LvgA, which is part of the complex that recognises effector proteins to be exported by their type IVB secretion system. Kim et al. (2020) describe this protein as an adaptor, recruiting multiple other effector proteins to the complex.

Baron et al. (2007) also identified CARP4, the only CARP without localization information on TrypTag.org (accessed 02/04/2023), as a flagellar protein. CARP4 is orthologous to *Chlamydomonas reinhardtii* Rib72 (Chlre5_6|10141; BLASTP E-value = 3.1E-28), which appears to be involved in linking microtubules of the outer doublets of the axoneme (Ikeda et al., 2003). Like CARP4, CrRIB72 contains three DM10 domains and a C-terminal EF-hand domain, which binds Ca^2+^ and is implicated in regulatory and signalling responses (Nelson et al., 2002). Moreover, CARP4 is orthologous to the human EF Hand Containing protein EFHC1 (BLASTP E-value = 5E-55) that is linked to Juvenile Myoclonic epilepsy (King, 2006) and is believed to be part of the structure of cilia (King, 2006; Suzuki et al., 2020). Structurally, CARP4 aligned with other EF Hand domain-containing proteins, specifically the Ca^2+^ sensor Case16 (Leder et al., 2010) and an EF-hand-containing domain of a spectraplakin that cross-links microtubule and actin and binds two Ca^2+^ (Lane et al., 2017). It is thus likely that CARP4 is a Ca^2+^-binding protein involved in direct interactions between flagellar microtubules and, possibly, other effector proteins.

CARP9 is annotated as Component of Motile Flagella 19 (CMF19) in TriTrypDB. It contains a RIB43A domain, a structure that was first identified in *Chlamydomonas*, and is associated with protofilament ribbons and basal bodies of ciliary and flagellar microtubules (Norrander et al., 2000). As the microtubular ribbons in *Chlamydomonas* mostly consist of RIB43A and RIB72 in addition to tubulin (King, 2006), it is likely that CARP4 and CARP9 play a similar role in *T. brucei*, with the EF-hand domain providing Ca^2+^-mediated regulation. Curiously, CARP9 was co-purified with the *T. brucei* Mitochondrial Calcium Uniporter (TbMCU; (Huang and Docampo, 2020)), although it is not immediately clear how a putative axonemal protein and an integral membrane protein of the inner mitochondrial membrane might associate *in vivo*. The CARP9 structure consists of two long alpha-helical rods, connected by a third, inter-strand helix (alphafold.ebi.ac.uk/entry/Q57UY7) and structural similarity searches were not very informative regarding a specific function. CARP6 also contains an EF-hand domain (**Figure 4**), reinforcing the link to Ca^2+^ signalling somewhere in the response to high cAMP; it is structurally related to calmodulin (Ishida et al., 2000).

CARP3’s predicted structure shows a highly structured N-terminus essential for localization of the protein at the distal tip of the flagellum in procyclic trypanosomes (Bachmaier et al., 2022) where it interacts with several adenylate cyclases and regulates social motility and tsetse fly transmission (Bachmaier et al., 2022; Shaw et al., 2022). Interestingly, the closest structural match for CARP3 was *Bacillus subtilis* RapH, a phosphatase response regulator (Parashar et al., 2011), which is consistent with a regulatory role in the cAMP response.

CARP10 contains a series of ten predicted Membrane Occupation and Recognition Nexus (MORN) domains as well as two copies of the Histone H3 K4-specific methyltransferase SET7/9 N-terminal domain superfamily (101-220 and 201-300). Although the MORN domain has been identified in a total of 113,928 proteins in many species (SMART database; http://smart.embl.de/smart/; 25/07/2022), the function of this domain is still poorly understood (Zhou et al., 2022). However, the structure of the domain in CARP10 is closely related to *H. sapiens* MORN4, which Li et al. (2019) describe as containing an extended single-layered β-sheet structure that uses a U-shaped groove to bind the long α-helix tail of myosin 3A. CARP10, being localised to the axoneme based on GO terms and TrypTag and sharing the U-shaped β-sheet (‘long open tube’) structure of HsMORN4, could therefore be implicated in coupling regulatory proteins to the flagellar motor function.

Finally, cAMP binding motifs were identified in the sequences of two CARP proteins, CARP1 and CARP11, i.e. the genes to which the highest and the lowest percentage of reads, respectively, were mapped in the RIT-seq screen. In CARP1, three cAMP binding domains are detected at a.a. positions 233–346, 370–491 and 519–577, 612–651, whereas in CARP11 a single cyclic nucleotide binding domain is detected, with insertions, at positions 293–358, 415–437 and 519–545.

**Figure 5:**
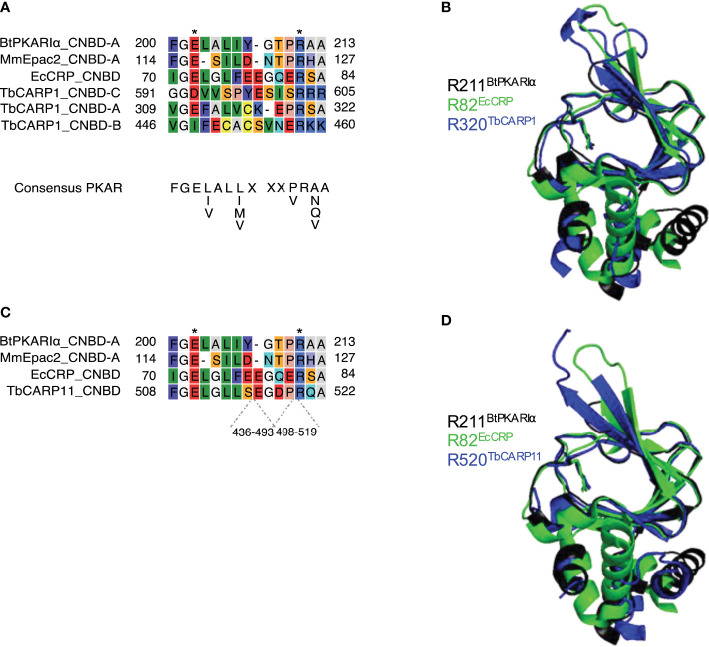
Sequence comparisons **(A, C)** of the predicted phosphate binding cassettes (PBCs) of *T. brucei* CARP1 (A, CNBD-A, -B, -C) or CARP11 **(C)** with *Escherichia coli* CRP (EcCRP), *Mus musculus* EPAC2 (MmEpac2) and *Bos taurus* PKARIα (BtPKARIα). The amino acid numbers of the predicted sequence insertions within the CARP11 PBC are indicated below the alignment. Asterisks indicate conserved Glu and Arg residues known to be essential for cAMP binding (Berman et al., 2005). **(B, D)** Structural alignments of CARP1 CNBD-A (B, dark blue) or CARP11 CNBD (D, dark blue) with BtPKARIa CNBD-A (PDB 1RGS, black) and EcCRP CNBD (PDB 4N9H, green) with the conserved arginines R320 of CARP1 and R520 of CARP11, respectively, overlaying the conserved arginines R211 of BtPKARIa CNBD-A and R82 of EcCRP CNBD. The structures were retrieved from http://wheelerlab.net/alphafold/ and superimposed with Pymol version 2.5.4.

The putative phosphate binding cassette (PBC) sequences of the predicted CARP1 and CARP11 cAMP binding domains were aligned with well-characterised but diverse cAMP binding domains from different species: *E. coli* CRP (EcCRP, Uniprot P0ACJ8), *Mus musculus* EPAC4 (MmEpac4, Uniprot Q9EQZ6) and *Bos taurus* PKARIα (BtPKARIα, Uniprot P00514) in **Figure 5**. The structural searches also turned up diverse cyclic nucleotide binding domains for both CARPs, including the regulatory subunit of *Toxoplasma gondii* PKA as the top hit for CARP11 (El Bakkouri et al., unpublished; www.rcsb.org/structure/5J3U). A PBC consensus sequence that was compiled for PKA regulatory subunits, F-G-E-[LIV]-A-L-[LIMV]-x(3)-[PV]-R-[ANQV]-A (Canaves & Taylor, 2002; Kannan et al., 2007), is also displayed for comparison. Highly conserved residues of the PBC include an invariant arginine (R211 in BtPKARIα) known to interact with the phosphate group of cAMP and a glutamate (E202 in BtPKARIα) that interacts with the 2’OH group of the ribose (Berman et al., 2005). While CNBD-A of CARP1 and CARP11 match very well with the PKA consensus, CNBD-B and -C of CARP1 have important deviations and the validity of the alignment and cAMP binding prediction are questionable (**Figures 5A, C**). Structural alignment of the CNBDs in the AlphaFold2-predicted CARP1 structure (retrieved from http://wheelerlab.net/alphafold/; Wheeler, 2021) with published crystal structures of diverse cAMP binding proteins (EcCRP, PDB 4N9H; BtPKARIα, PDB 1RGS) showed a very good structural preservation including overlay of R320 in CARP1 CNBD-A with the conserved arginines of the reference protein structures (**Figure 5B**, RMSD 1.168 with EcCRP CNBD; RMSD 0.782 with BtPKARIα CNBD-A). The same observation is made for R520 in the CARP11 CNBD (**Figure 5D**, RMSD 1.039 with EcCRP CNBD; RMSD 0.602 with BtPKARIα CNBD-A). In contrast, the conserved arginine in the reference structures aligned with Lys459 and Tyr629 in CNBD-B and CNBD-C of CARP1, respectively (**Supplemental Figures S1A, B**).

### 3.5 Experimental validation of cAMP binding of CARP1 and CARP11

**Figure 6:**
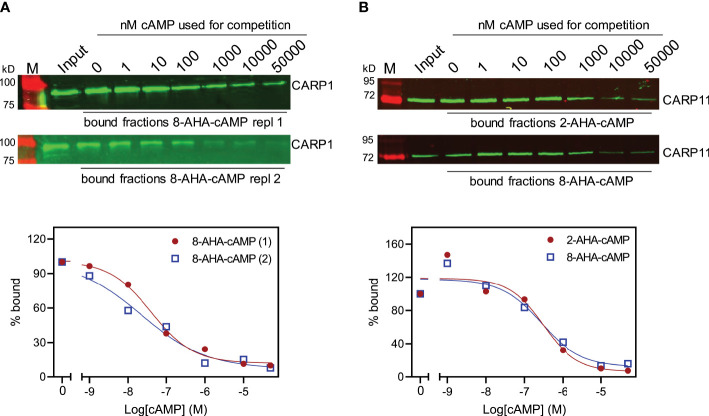
Cyclic AMP affinity purification of CARPs with predicted cAMP binding domains. **(A)** CARP1 was pulled down by 8-AHA-cAMP agarose from *T. brucei* cells overexpressing CARP1 (2 replicates). **(B)** CARP11 was expressed in *E. coli* with an N-terminal His_6_-SUMO tag and pulled down by 2-AHA- (top) or 8-AHA-cAMP agarose (bottom). The Western blots show the input fractions and the pulled down material in presence of increasing concentrations of cAMP (0 nM – 50 µM) for competition. The load of the bound fractions relative to the input is 15× for CARP1 and 12.5× for CARP11. Binding curves from the fluorescence quantifications are shown below; the amount of CARP pulled down without competition was set to 100%.

In order to test whether CARP1 and CARP11 do bind cAMP, we performed a pull-down experiment with cAMP immobilised on agarose beads *via* a flexible aminohexyl linker on position 8 (8-(6-aminohexylamino)-adenosine-3’, 5’-cyclic monophosphate; 8-AHA-cAMP-agarose). Lysates of *T. brucei* overexpressing CARP1 were first incubated with agarose beads without attached cAMP in order to remove proteins non-specifically binding to the bead matrix. Subsequent incubation with 8-AHA-cAMP-agarose allowed the pulldown of a fraction that was highly enriched in cAMP-binding proteins. This procedure was performed in the presence of increasing cAMP concentrations (0 – 50 µM) intended to outcompete the binding to 8-AHA-cAMP. Subsequent elution of the protein fraction from the agarose beads, followed by Western blot with a rabbit anti-CARP1 serum gave a quantifiable amount of CARP1 that was plotted against the cAMP concentration to derive an approximation of cAMP binding affinity. The antiserum had been validated using a panel of *T. brucei* cell lines, showing an increased signal in the CARP1-overexpressing strain and a strong decrease upon RNAi knockdown (**Supplemental Figure S2A**). **Figure 6** shows the average of two such determinations plotted with a sigmoidal curve with variable slope (standard inhibition; r^2 ^= 0.977) yielding an EC_50_ value of 32.4 nM (95% CI 14.6 – 76.1 nM). Approximately 0.6–7.5% of the input CARP1 was pulled down in the competition series from 0 to 50 µM. Most likely this represents the active, properly folded fraction of the highly insoluble CARP1. A further pull-down with cAMP-agarose beads was performed using a set of differently linked cAMP analogues. Beads with various linkers connected to the purine ring at positions 2, 6, or 8 all showed similar capacity to pull down CARP1, but two different linkers attached to the 2’ position of the ribose failed to pull down any CARP1 (**Supplemental Figure S3A**), consistent with cAMP interaction within the CNB pocket primarily *via* the ribose and cyclic phosphate.

The same procedure was performed with CARP11 expressed with a His_6_-SUMO tag in *E. coli* (**Figure 6**), using 8-AHA-cAMP and 2-AHA-cAMP agarose beads. For Western blots, a mouse anti-His serum was used for detection of the tagged CARP11. The competition titrations with cAMP in **Figure 6** show similar binding to 8-AHA-cAMP and 2-AHA-cAMP agarose beads with almost identical EC_50_ estimates: 309 nM for 2-AHA-cAMP and 276 nM for 8-AHA-cAMP. Approximately 0.6–24.5% of the input CARP11 was pulled down in the competition series from 0 to 50 µM (**Supplemental Figure S3B**). We conclude CARP1 and CARP11 are cAMP-binding proteins and that CARP1 has a ~10× higher affinity for the cyclic nucleotide than CARP11.

## 4 Discussion

By genome-wide RNAi screening for cAMP resistance in bloodstream stage trypanosomes, we have identified a set of proteins with a predicted function in cAMP metabolism or cAMP signalling. The selection was based on the essentiality of cAMP metabolism (Oberholzer et al., 2007; Shakur et al., 2011; Salmon et al., 2012a) and a strong cell division phenotype resulting from genetic or pharmacological perturbations of cellular cAMP concentrations (de Koning et al., 2012; Salmon et al., 2012a; Gould et al., 2013). Strikingly, for all identified cAMP response proteins (CARPs) we found publicly available experimental evidence (summarized in **Figure 4**) for association with the flagellar axoneme or the flagellum, suggesting an important impact of cAMP signalling on flagellar biology. An intact and functional flagellum was previously shown to be essential for cell division of bloodstream stage trypanosomes, as RNAi repression of many genes with flagellar localization or function result in a lethal cell division phenotype (Broadhead et al., 2006; Marques et al., 2022). This may explain why the screen returned specifically the observed set of proteins. Initiation of flagellar replication is the necessary first step in trypanosome cell division (Vaughan, 2010; Sun et al., 2013), and impaired flagellar function results in a block in cytokinesis. This is exactly the lethal phenotype seen with non-physiologically elevated cAMP (de Koning et al., 2012; Gould et al., 2013). Consequently, cyclic nucleotide phosphodiesterases (PDEs) of trypanosomes are considered good drug targets (Blaazer et al., 2018; de Heuvel et al., 2019).

A priori, we expected to get from our RNAi screening the following protein categories (1) proteins involved in cAMP production like adenylate cyclases (ACs) or proteins affecting the activity or abundance of ACs, (2) effectors binding cAMP and (3) a diverse group of targets downstream of the effectors or modulating the activity of those effectors. As expected, the hit list included several AC genes, but at relatively low RIT-seq representation. The *T. brucei* genome contains more than 70 receptor-type ACs, the catalytic domains of which share homology with mammalian class III cyclases (Pays & Nolan, 1998; Salmon et al., 2012b; Salmon, 2018; Durante et al., 2020). Many of the *T. brucei* ACs have been shown to be developmentally regulated (Saada et al., 2014; Naguleswaran et al., 2018), while others are expressed throughout the life cycle (Alexandre et al., 1996). The fact that we observed only relatively few ACs, and at relatively low RIT-seq representation (**Figure 1**), may be explained by the limited impact on the cAMP concentration upon RNAi of any individual AC family members (Salmon et al., 2012a). A higher RIT-seq representation was found for CARP3, a membrane-associated protein that interacts with several ACs, including the ESAG4 cyclase that is abundant in bloodstream forms, and positively regulates their abundance (Bachmaier et al., 2022). It is therefore likely that CARP3 repression confers cAMP resistance in bloodstream forms by concomitant downregulation of a significant fraction of ACs, particularly the dominant ESAG4, and thereby lower cAMP production.

At the effector level, we have identified two novel cAMP-binding proteins from the RNAi screen, CARP1 (Gould et al., 2013) and CARP11. The *T. cruzi* CARP1 homolog (TcCLB.508523.80) also seems to bind cAMP (Jäger et al., 2014). CARP1 has three predicted CNB domains; the phosphate binding cassettes (PBCs) of the first (CNBD-A) and of the CNB domain of CARP11 match a consensus sequence for the PBCs of PKA of diverse species very well (**Figure 5A, C**). In contrast, CNBD-B and CNBD-C of CARP1 deviate in critical residues and therefore, we can neither predict nor exclude their contribution to cAMP binding in our immobilised cAMP assay (see also **Figure S1**). Interestingly, CARP11 has two longer sequence insertions within the CNBD that apparently do not compromise the cAMP binding function. AlphaFold2 modelling of the domain and overlay with reference CNB crystal structures (**Figure 5D**) show indeed a well conserved CNB structure in CARP11, suggesting that the insertions have accessory functions compatible with cAMP binding. The binding affinity, estimated by EC_50_ of cAMP competition, is ~30 nM for CARP1, a value in the range of the free regulatory subunit of PKA from other organisms (Moll et al., 2007). The EC_50_ for CARP11 is ~300 nM and closer to effectors like mammalian EPAC (Dao et al., 2006) and to physiologically relevant cAMP concentrations in cAMP microdomains (Anton et al., 2022).

The remaining seven CARP genes identified in the genome-wide RNAi screen cannot be assigned yet to a specific biochemical function. Some of them are most likely targets downstream of the cAMP signal, supported by the identified domains and available data on potential orthologs in higher eukaryotes (e.g. CARPs 2, 4, 9). For others (CARPs 6, 7, 8, 10), a function at the level of cAMP signal production, as found for CARP3, is equally possible. EF-hand domains (Nelson et al., 2002) in CARP4 and 6 suggest crosstalk between cAMP signalling and regulatory processes involving Ca^2+^.

Most importantly, the experimentally determined subcellular localization in trypanosomes retrieved from the database provided by the TrypTag project (Dean et al., 2017; Billington et al., 2023) assigned 7/10 CARPs to the flagellar axoneme and 2/10 (CARPs 2 and 4) were assigned to the flagellum based on proteome data (Broadhead et al., 2006; Subota et al., 2014). CARP3 is associated with the flagellar membrane and the axonemal cap *via* FLAM8 (Bachmaier et al., 2022). Thus, all CARPs are predicted to have a role in flagellar biology. By inference, cAMP signalling is important for regulation of flagellar functions. The impact is not limited to trypanosomes: 5/7 of the CARPs present in the *Leishmania* genome (**Figure 3**) have been found enriched in the flagellar insoluble fraction by proteome analysis (CARPs 2, 4, 6, 9, 10 (Beneke et al., 2019)). The CARPs thus provide a valuable resource to functionally dissect important flagellar biology of kinetoplastid parasites. Indeed the longer list of 44 genes with normalised RIT-seq mapped reads ≥ 0.05 (**Supplemental Table S2**) was highly enriched with axonemal (16/44) and other flagellar (7/44) localizations (TrypTag.org) and was significantly enriched for the GO component terms ‘axoneme’ (6-fold; P = 2.7×10^-8^), ‘ciliary plasm’ (2.3-fold; P = 7.7×10^-5^) and ‘cilium’ (1.85-fold; P = 5.7×10^-4^). One potential entry of interest is hypothetical protein Tb927.7.4100, which encodes a 500-a.a. protein that like CARP7 features a DUF4201 domain and is located to the flagellum by proteomic analysis (Subota et al, 2014) and to the axoneme by TrypTag. This dataset could thus provide a starting point for further investigation into flagellar biology as well as novel cAMP-regulated pathways essential for parasite growth, survival and transmission. These pathways are potential targets for drug development against kinetoplastid diseases.

One example of an in-depth analysis is provided by CARP3. This protein localizes to the flagellar membrane in bloodstream forms but is restricted to a specialised microdomain at the tip of the flagellum in the procyclic stage (Bachmaier et al., 2022). In that stage, cAMP signalling *via* tip-localized adenylate cyclases (ACs) has been shown to regulate social motility “SoMo” on agarose plates (Lopez et al., 2015; Oberholzer et al., 2015), a phenotype that is now interpreted as chemotaxis along a pH gradient towards a more basic environment (Shaw et al., 2022). CARP3 interacts with multiple ACs in a flagellar tip complex and is essential for “SoMo” and for colonization of tsetse fly tissues and thus transmission (Bachmaier et al., 2022; Shaw et al., 2022; Shaw & Roditi, 2023). CARP3 differentially regulates the abundance of ACs, and a model predicts that it controls the relative abundance of functionally different cyclases for integration of various external signals (Bachmaier et al., 2022). The flagellar tip and membrane localization of CARP3 was also seen in *T. cruzi* (Won et al., 2023). The specific role in signalling from the environment to the cell, e.g. pH chemotaxis, is consistent with the membrane-associated localization of CARP3. In contrast, the other CARPs are localized to the axoneme and therefore more likely play roles in cell autonomous pathways, regulating flagellar motility. The fact that flagellar motility *per se* is unaffected in CARP3-depleted SoMo-negative cells (Bachmaier et al., 2022) also argues for distinct pathways. In mammalian cells, cAMP is involved in regulating ciliar motility within the axoneme (Wirschell et al., 2011) as well as distinct cilial sensing pathways (Nachury & Mick, 2019).

The novel cAMP-binding protein CARP1 appears in the genome of *Euglenozoa* (**Figure 3**), also supported by detection of CARP1, 4, 6, 8, 9, 10 in the flagellar proteome of *Euglena gracilis* (Hammond et al., 2021). In contrast, CARP11 is only detected in *Trypanosoma*. We notice a striking correlation between the exclusive presence of these cAMP effectors in *Euglenozoa* and the absence of a cAMP-dependent protein kinase A. PKA in *T. brucei* and *T. cruzi* is structurally conserved but repurposed for binding and regulation by nucleoside analogs (Bachmaier et al., 2019). Future dissection of the novel flagellar cAMP signalling mechanisms may indicate why repurposing of PKA for a different second messenger or activation mechanism might have conferred a selective evolutionary advantage to organisms with novel cAMP effectors.

## Data availability statement

The datasets presented in this study can be found in online repositories. The RIT-seq data was submitted to the European Nucleotide Archive (https://www.ebi.ac.uk/ena/browser/home), with submission number PRJEB60531.

## Author contributions

MB, DH and HK designed and supervised research; SB, MG, EP, RO, AB, MA, JM performed research; SB, MG, HK, MB and DH analyzed data; HK, MB and SB wrote the paper. All authors contributed to the article and approved the submitted version.
