# Supplementary material for: Novel kinetoplastid-specific cAMP binding proteins identified by RNAi screening for cAMP resistance in Trypanosoma brucei
Source: Front Cell Infect Microbiol. 2023 Jul 5;13:1204707. doi: 10.3389/fcimb.2023.1204707 (PMC10354285; doi:10.3389/fcimb.2023.1204707)
Supplement: Supplementary file 1 [file DataSheet_1.docx]

Supplementary Material

Novel kinetoplastid-specific cAMP binding proteins identified by RNAi screening for cAMP resistance in *T. brucei*

Sabine Bachmaier, Matthew K. Gould, Eleni Polatoglou, Radoslaw Omelianczyk, Ana E. Brennand, Maha A. Aloraini, Jane C. Munday, David Horn, Michael Boshart^*^, Harry P. de Koning^*^

*** Correspondence:** Michael Boshart, [boshart@lmu.de](mailto:boshart@lmu.de); Harry P. de Koning, [Harry.De-Koning@glasgow.ac.uk](mailto:Harry.De-Koning@glasgow.ac.uk)

## Supplementary Figures


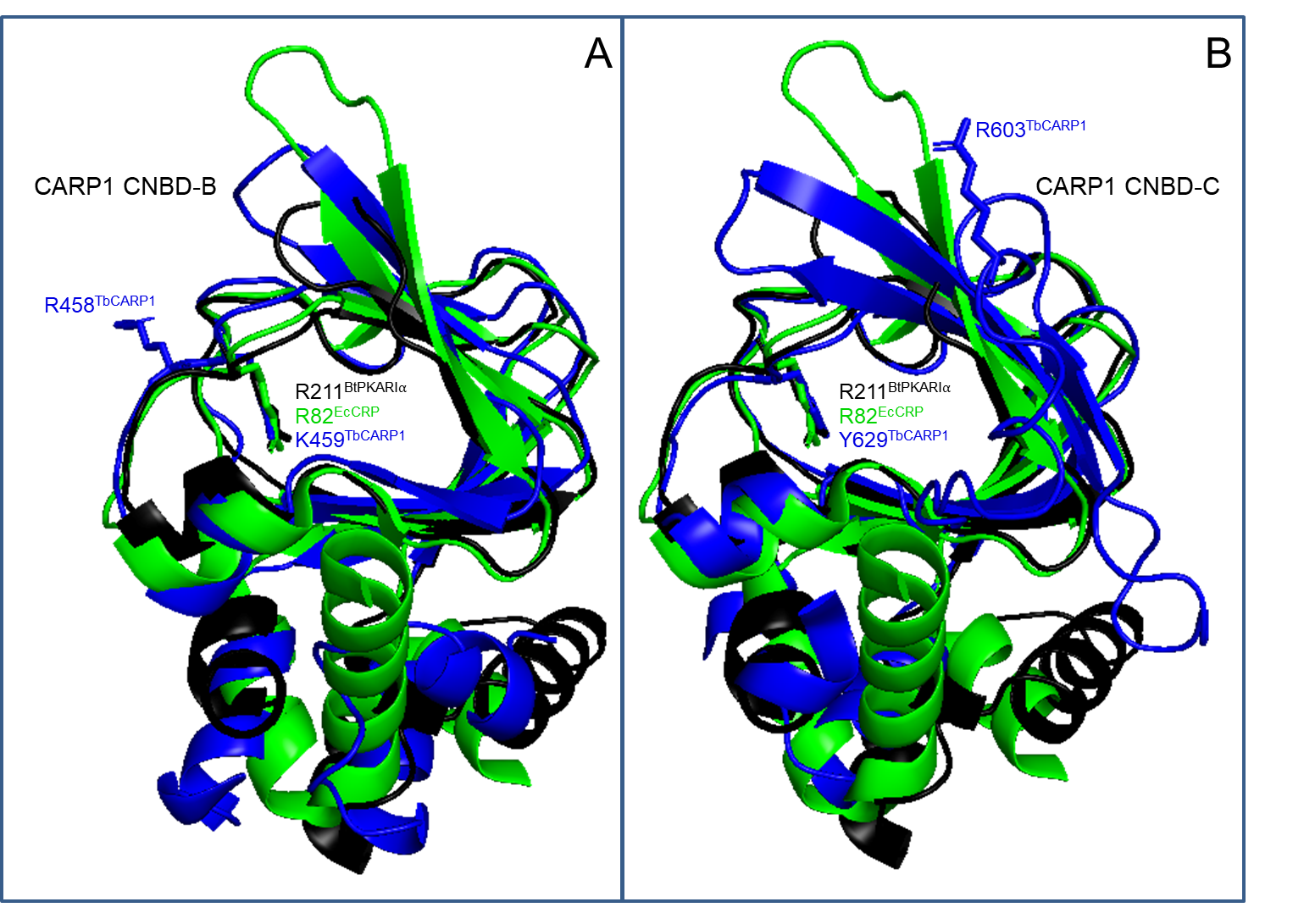


**Supplementary Figure 1.** Structural alignment of CARP1 CNBD-B (A) and CNBD-C (B) with BtPKARIa_CNBD-A (PDB 1RGS, black; conserved R211 sidechain shown) and EcoliCRP_CNBD (PDB 4N9H, green; conserved R82 sidechain shown). The CARP1 CNBDs are coloured in blue. In addition to the sidechain of the Arg that is the best alignment fit in Fig. 5A, the residues that structurally align with R211^BtPKARI⍺^ and R82^EcCRP^ are displayed. The CARP1 structure predictions were retrieved from <http://wheelerlab.net/alphafold/> and superimposed with Pymol version 2.5.4.

**
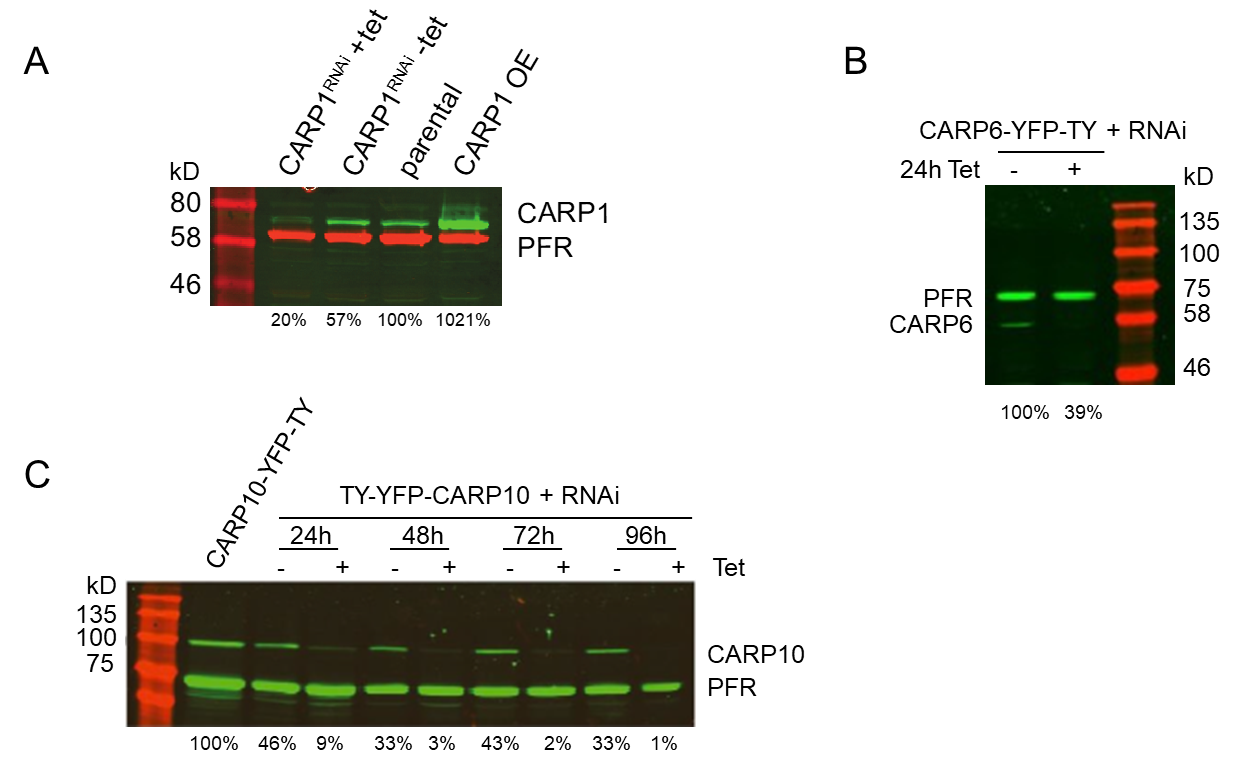
Supplementary Figure 2.** (A) Specificity of the affinity-purified rabbit anti-CARP1 antibody by Western blot analysis. CARP1 expression in trypanosomes was regulated by tetracycline-inducible *CARP1* RNAi (+tet/-tet, 24h) or by CARP1 overexpression (OE) relative to the parental line. CARP1 signals (green) were normalized to the PFR-A/C loading control (red) and the normalized signal in the parental cells was set to 100%. (B) Control of knock-down efficiency for CARP6 upon tetracycline-inducible RNAi in a cell line expressing *in situ* tagged CARP6-YFP-TY. CARP6-YFP-TY signals detected by anti-TY were normalized to the PFR-A/C loading control. Relative intensity values are given below the Western blot. (C) Control of knock-down efficiency for CARP10 upon tetracycline-inducible RNAi in a cell line expressing *in situ* tagged TY-YFP-CARP10. TY-YFP mean a TY Tag N-terminal to a yellow fluorescent protein. Detection and quantification as in (B). PFR = paraflagellar rod proteins A and C (antibody recognises both).

**
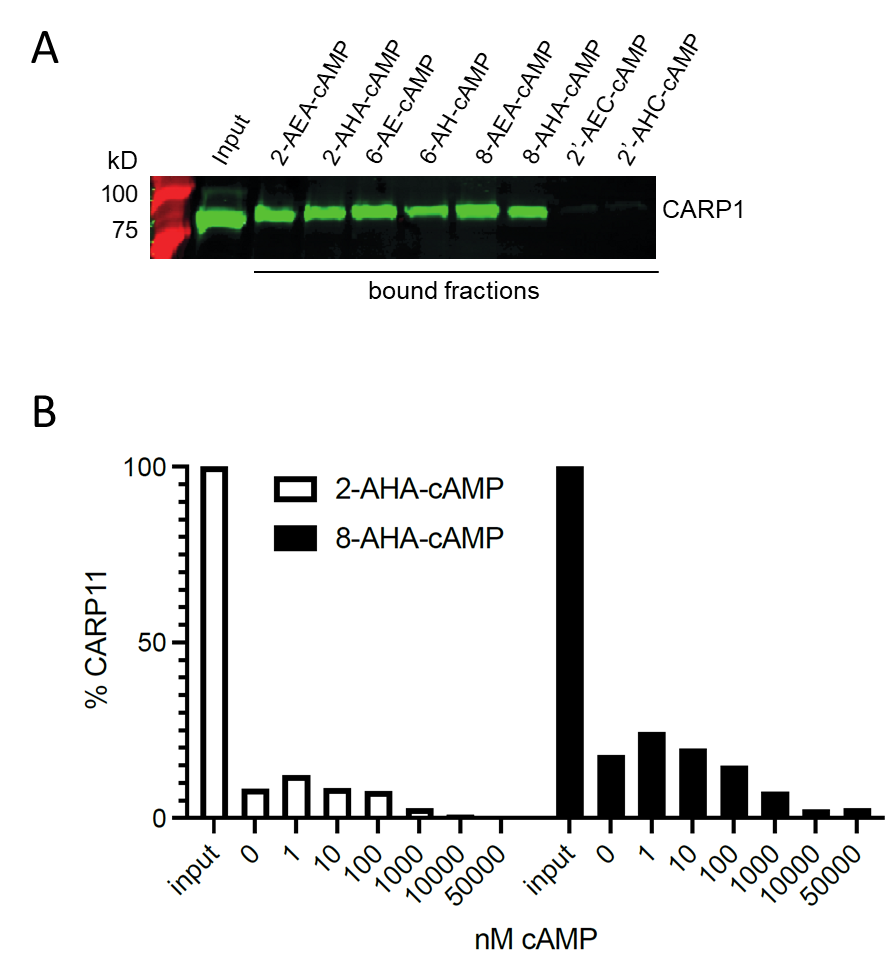
**

**Supplementary Figure 3.** (A) CARP1 pull-down from *T. brucei* cells overexpressing CARP1 with cAMP agarose beads with different linkers as indicated. The Western blot shows the input fraction and the pulled down material (bound fractions) and was probed with rabbit anti-CARP1 (green). (B) CARP11 pull-down from *E. coli* with 2-AHA- or 8-AHA-cAMP beads with bound CARP11 shown as % of the input (set to 100%) in presence of increasing concentrations of cAMP (0 nM – 50 µM) for competition. Plotted values are derived from the blots shown in Fig. 6B.
